# Cleaning Products, Environmental Awareness and Risk Perception in Mérida, Mexico

**DOI:** 10.1371/journal.pone.0074352

**Published:** 2013-08-23

**Authors:** Ruth Magnolia Martínez-Peña, Almira L. Hoogesteijn, Stephen J. Rothenberg, María Dolores Cervera-Montejano, Julia G. Pacheco-Ávila

**Affiliations:** 1 Independent researcher, 113 Woodview Terrace, Hurst, Texas, United States of America; 2 Centro de Investigación y de Estudios Avanzados del Instituto Politécnico Nacional, CINVESTAV, Departamento de Ecología Humana, Unidad Merida. Km. 6 Antigua Carretera a Progreso, Mérida, Yucatán, México; 3 Instituto Nacional de Salud Pública, INSP, Universidad No. 655 Colonia Santa María Ahuacatitlán, Cerrada Los Pinos y Caminera, Cuernavaca, Morelos, México; 4 Universidad Autónoma de Yucatán, UADY, Facultad de Ingeniería, Av. Industrias no contaminantes por Periférico Norte Apartado Postal 150, Cordemex, Mérida, Yucatán, México"

## Abstract

Cleaning products are associated with many health and environmental problems. Contamination of water resources by cleaning products is more likely to occur with septic tanks as sewage treatment systems especially in karstic terrains. We explored women’s ideas about water sources and the risk cleaning products pose to health and sewage in Mérida, a city in the Yucatán peninsula of Mexico. Women were unaware of the city’s water management system. We found a positive and statistically significant association between risk perception and environmental awareness, education level and employment status. We suggest developing education and risk communication strategies to inform residents about the hydro-geological features in the Yucatán, the vulnerability of its karstic aquifer and the health and environmental risks associated with cleaning agents.

## Introduction

Although cleaning has been a common activity in an historical context, it is since the 19th century that complex mass marketed cleaning products form part of our cleaning habits [1]. During the last century thousands of different cleaning products have been produced, generating phenomenal global growth in the industry [2]. Advertising of cleaning products has been mostly directed at women and is supported by the idea that cleaning is synonymous with happiness [3] and health. Nonetheless, studies have identified the adverse consequences of cleaning products on human health [4] and the environment [5]. Contact dermatitis [6], asthma [7] and other respiratory disorders [8], poisoning [9] and bacterial resistance [10] have been associated with the use of cleaning products. Endocrine disruption has also been associated with exposure to cleaning products [11]. Adverse consequences in the environment include eutrophication [12], anatomical and physiological changes in aquatic and terrestrial fauna [13], the elicitation of estrogenic response in mammals and fish [14], the facilitation of the adsorption of pharmaceutical compounds such as acetaminophen and carbamazepine by aquifer materials and sediments [15], and the creation of water-repellent soils due to irrigation with gray water which contains surfactants [16].

Nearly 12% of the global land surface is formed by carbonate rocks, prone to chemical dissolution, i.e. karstification [17]. Karstic aquifers are the source of drinking water for about 25% of the world population [18], in both, developed [19] and developing countries [20]. Karstic aquifers are vulnerable to contamination [21], particularly from septic tank leachate [22]. In the United States, where about 20% of total households use septic tanks [23], boron [24] and phosphorus [25] derived from detergents were found in aquifers contaminated by septic waste in karstic terrain.

Popular awareness of the vulnerability of fresh water resources and concerns about the role played by the detergents in water pollution and the emergence of health problems can be explored through the concepts of Environmental Awareness (EA) and Risk Perception (RP). EA is the information that people have about any phenomena related to their environment [26,27], their concern about the environment [28], and their willingness to act in favor of the environment, including the behavior derived from that commitment [29]. The concept of EA has been used to analyze popular knowledge, concerns and attitudes about issues such as household electronic devices [30], water pollution [31], conservation [32] and climate change [33]. The RP concept functions in the context of the evaluation of judgments about activities and technologies considered to be risky [34]. RP studies have analyzed popular knowledge, attitudes and behaviors related to water reuse [35], pesticides [36], loss of agro-biodiversity [37] and West Nile virus [38]. When the object of the RP study is a product, RP refers to how much that people feel that their safety is threatened by using it [39]. To our knowledge there are no published studies relating cleaning products, RP and EA in the developing world in general and Mexico in particular, and cleaning products contamination studies are scarce. Detergent compounds have been found in the San Juan River in the state of Nuevo León [40], in the Texcoco River in central Mexico [41] and in the Lerma–Santiago River in the state of Mexico [42]. In the Yucatán Peninsula, high concentrations of phosphates associated with household and hotel sewage have been found in the coastal lagoons of Chelem in Yucatán and in Nichupte and Bojórquez in Quintana Roo [43].

Mérida’s karstic surface [44] was formed during the Pliocene-Miocene epoch [45]. The permeability of the karstic formation facilitates infiltration [46], increases groundwater vulnerability to pollution [47] and explains the lack surface water [48]. The aquifer is the only source of fresh water in the zone and its availability in the entire state is affected by saltwater intrusion, overexploitation [49] and by contamination [50]. In the urban area, water from the top 20 meters of the aquifer is not suitable for human consumption anymore [51]. As Mérida lacks a conventional sewerage system [52] untreated effluents infiltrate the groundwater [53]. Sewage management in Mérida relies upon septic tanks, most of which are not built to proper technical specifications [54,55] and thus hinder wastewater transformation, chemical degradation and biodegradation processes [56]. The aim of this paper is to analyze the answers of 739 women in Mérida, the capital city of the state of Yucatán (Mexico), which explored their knowledge of water sources and sewage treatment in the city and the effect of environmental awareness on perceived environmental and health risks related to household cleaning products. This study supplies information to risk communicators and health and environmental authorities applicable not only in Mérida, but in every city with karstic aquifers and on-site sewage disposal systems around the world.

## Materials and Methods

### Study site

Mérida is situated in the southeast of Mexico, between 20°41’ N and 21°12’ N, and 89°27’ W and 89°49’ W, at an altitude ranging from 7 and 10 m. a. s. l. [57]. Mérida has 830,732 inhabitants and covers approximately 88,300 ha (218,194 acres) [58].

### Sampling design

Mothers of children attending any of the six levels of primary school were invited to participate. Two criteria led to the focus on this population: the emphasis of cleaning product advertisements on the role of women in cleaning activities [59], and the vulnerability of children under 12 to poisoning by cleaning products [60]. Six public schools and 14 private schools were selected based on geographic distribution (north, center and south of the city). This selection was important given the socioeconomic segregation of the city, in which people of high income live to the north site and people of low income live in the southern site [61]. A total of 739 surveys were completed, 294 in public schools and 445 in private schools. 599 surveys were answered in school facilities and 140 were sent to the respondents’ homes and collected at the school one week later. We divided our survey in three sections: Environmental Awareness Scale (EAS, Table 1), Risk Perception Scale (RPS, Table 2) and sociodemographic data (age, education, income and occupation). EAS had 10 items with three possible answers: True, False and Not sure. RPS was a Likert scale with 16 items and five possible answers: Totally agree, Agree, Not sure, Disagree, and Totally disagree. A pilot version of the survey was conducted with 40 respondents, based on the results some changes were made to the final version. The surveys were performed between August and November 2007.

**Table 1:** Sociodemographic characteristics (control variables) of the study group.

	Data included in OLS		Data excluded from OLS		
Variable	N	%	N	%	*p* ^*^
Age					0.920
≤ 29	133	19.6	6	16.2	
30–44	491	72.4	28	75.7	
≥ 45	54	8.0	3	8.1	
Education level^**^					0.604
Low	263	38.8	11	47.8	
Middle	142	20.9	5	21.7	
High	273	40.3	7	30.4	
Income^***^					0.965
≤ 4	221	39.6	10	43.5	
>4 − 10	111	19.9	5	21.7	
>10 − 25	177	31.7	7	30.4	
>25	49	8.8	1	4.4	
Occupation					0.629
Housewife	382	56.3	20	60.6	
Outside employee	296	43.7	13	39.4	

^*^ p-values were calculated from Fisher exact and chi-squared tests, depending on the observed value in the cells.

^**^ Low: no educated-secondary school; middle: incomplete high school-incomplete college; and high: complete college-postgraduate.

^***^ Income is expressed as multiples of the minimum monthly wage in 2007 (MXN $ 1,428 or USD$116.4). Income data were reported only by 581 participants.

**Table 2:** Rasch Model Fitness diagnoses for the Environmental Awareness Scale (EAS).

			Outfit		Answers (%)	
ITEM^*^	Difficulty (logits)	Standard error	MNSQ	Z	Right	Wrong
A septic tank is a container that transforms gray water into clean	-1.84	0.12	0.82	-1.2	86	2
water.						
The sludge from septic tanks is obtained and transformed into	0.88	0.09	0.83	-2.9	42	10
fertilizer.						
The ocean is the source of tap water.	-0.57	0.09	0.87	-1.9	68	7
The water extracted from wells comes from underground water	-0.56	0.09	0.95	-0.7	69	9
sources.						
Almost the 100% of gray water in the city leaks into the ground	-0.94	0.1	0.97	-0.3	74	6
and can contaminate the underground sources of water.						
The gray water from households is transformed into drinking	0.78	0.09	1.01	0.2	43	18
water in treatment plants.						
Rain is the source of tap water.	-0.67	0.09	1.04	0.5	70	5
The gray water from septic tanks goes into the ground.	-0.01	0.09	1.13	2.1	59	13
Tap water comes from underground water sources.	0.45	0.09	1.08	1.4	51	17
Sludge and gray water from septic tanks go to a hole on the	2.48	0.11	1.33	2.1	17	30
ground.						

^*^ Items are listed in order of MNSQ.

The research project and the techniques to obtain the data were approved by the Professors Board of the Human Ecology Department of CINVESTAV, according to Postgraduate Studies Regulations (Art. 43). In each school, a verbal authorization to send each child home with a written invitation for the mother to participate was provided by the general director. That invitation had the information about the date and the place where the survey would be conducted. It also stated the voluntary nature of this activity and the confidentiality to manage personal data. Once in the room, the women who attended the invitation where asked again about their intention to participate, and pictures of this process were taken. The fact that the women responded to the invitation and gave verbal recognition of their intent to participate confirmed their consent. Following the request of the directors of three of the private schools, the survey was attached to the invitation to give the women the opportunity to complete it at home. In this case, the consent was indicated by returning the completed survey.

### Statistical Analysis

The Rasch model [62] was used in order to process the ordinal data from EAS and RPS as continuous data, all assumptions were met. Rasch analysis is a probabilistic model based on the assumption that the measuring scale measures only one dimension or latent variable (EA or RP in our case) which can be used to rate the item’s difficulty as well as the subject’s ability [63]. Subjects and items share the same measurement scale in logits or the log-odds transformation of the probability of a response [64]. The scale to calibrate the difficulty of the items has zero (0) as the middle point, which means an equal likelihood to answer it correctly or incorrectly [65]. Zero is also the middle point of the scale to calibrate the ability of the subjects and means an equal likelihood to make a mistake or to have a correct answer [66]. The probability of a right or expected answer is a function of the difference between the difficulty of the item and the ability of the subject [67]. Unlike the classic approaches to measure abilities which emphasize fitting models to data, Rasch analysis emphasizes fitting data to the model [68]. Under the Rasch model, an item provides information about a subject’s performance when the quadratic mean of Outfit, MNSQ (an outlier sensitive fit statistic that picks up rare events), is between 0.5 and 1.5 [69].

Fitting of EAS and RPS to the Rasch model was analyzed with *Winsteps* software [70]. Fifteen surveys were omitted due to a lack of answers to the EAS items. All the EAS items (10) had MNSQ values between 0.5 and 1.5. The EA score was obtained for 724 participants. In the RPS analysis the original five possible answers were reduced to three by grouping Totally Agree and Agree into the Agree category, and Totally Disagree and Disagree into the Disagree category. Twenty- three surveys were not considered due to a total lack of answers in the RPS items. Two items had Outfit MNSQ higher than 1.5 and were removed from the final analysis of difficulty and ability. The RP score was obtained for 716 participants.

Prior to analysis, data were tested for normality using Skewness/Kurtosis, Shapiro–Wilk and Shapiro–Francia tests. Student’s t-tests were performed to compare EA and RP scores between groups with and without complete data in the sociodemographic section of the survey. To analyze the relationship between the dependent variable EA score and independent variables OLS regression analysis was performed, only with surveys containing complete data on age, education and occupation (n = 678). Independent variables considered were age (≤ 29, 30–44 and ≥ 45), education level (low: no educated–secondary school; middle: incomplete high school–incomplete college; and high: complete college–postgraduate) and occupation (housewife and outside employee). A standard errors adjustment was performed to avoid heteroscedasticity effects. To analyze the relationship between the dependent variable RP score and independent variables (EA score, age, education level and occupation) OLS regression analysis was performed. Income was considered a potential control variable in both models (EA and RP) but it was not included due to collinearity and low response rate. OLS regression was performed with a standard errors adjustment and 10000 bootstrap repetitions to avoid heteroscedasticity effects and the lack of residual normality.

The data were processed with *Access 2002* and *Excel 2002* [71], *Stata 10 2007* [72] and *Winsteps* [73].

## Results

**Figure 1:**
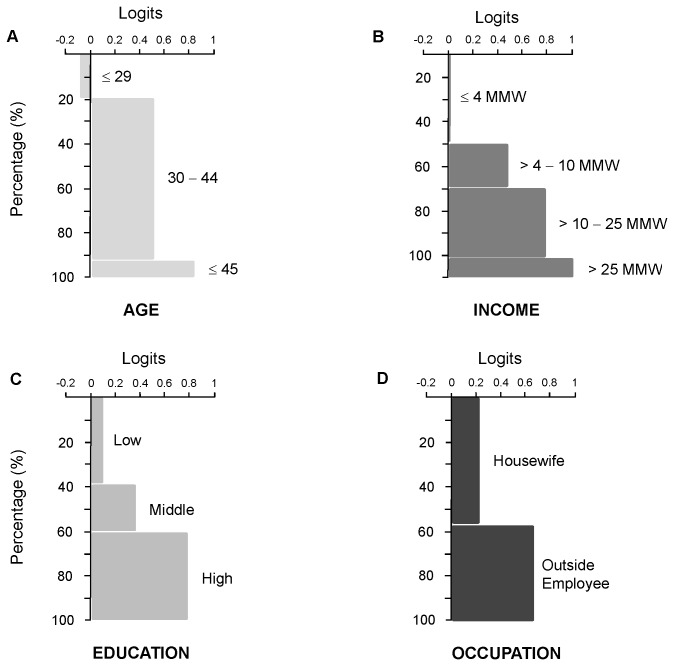
Mean of Environmental Awareness (EA) in logits, according to the sociodemographic variables. **A**. Age; **B**. Education level (low: no educated−secondary school; middle: incomplete high school−incomplete college; and high: complete college−postgraduate); **C**. Income (expressed as multiples of Minimum Monthly Wage, MMW. One MMW = USD $110); and D. Occupation.

No statistically significant differences were found between surveys completed at the school versus those completed at home. No significant differences were found between sociodemographic characteristics of participants who were included and those who were excluded from the OLS regression analysis (Table 1). The maximum and minimum difficulty values for the EAS items were 2.48 and -1.84, respectively, with zero as mean and standard deviation (SD) of 1.14 (Table 2). The maximum and minimum EA ability values were 4.16 and -3.97, respectively, with zero as mean and SD of 1.28. The mean of EA scores by sociodemographic variable and level is shown in Figure 1. The percentages of right and wrong answers to each EAS item are shown in Table 2.

About 70% of the participants were aware that rain and the ocean are not sources of tap water; 51% knew that the actual water source is groundwater (Table 2). More than 70% of the participants were not aware about the environmental fate of sludge and gray water; 43% of participants knew that the local treatment systems do not turn sewage water into drinkable water, and 42% knew that sludge is not used by the local authorities to produce fertilizers (Table 2). More than 70% of the participants were aware that the aquifer is vulnerable to contamination (Table 2).

**Figure 2:**
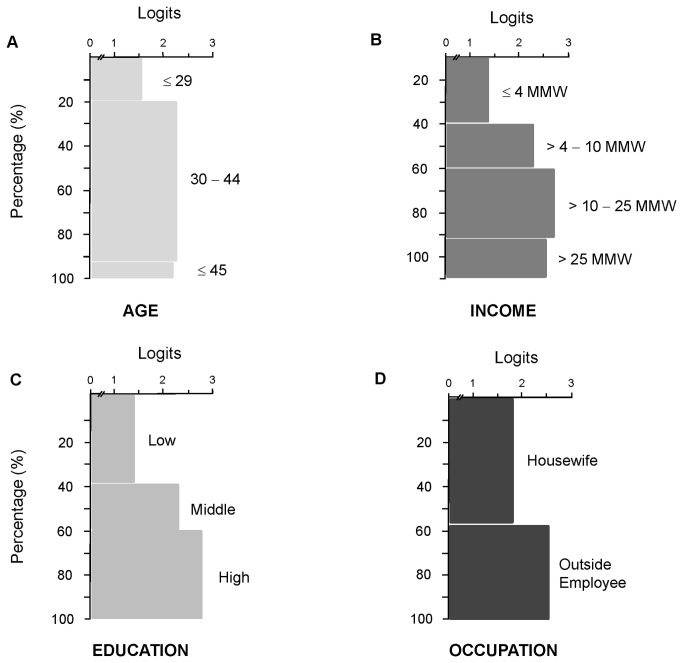
Mean of Risk Perception (RP) in logits, according to the sociodemographic variables. **A**. Age; **B**. Education level (low: no educated−secondary school; middle: incomplete high school−incomplete college; and high: complete college−postgraduate); **C**. Income (expressed as multiples of Minimum Monthly Wage, MMW. One MMW = USD $116); and D. Occupation.

Two items of the RPS were removed from the analysis due to their high difficulty values (5.61 y 3.65, respectively). The maximum and minimum difficulty values for the 14 items retained in the RPS were 1.35 and -3.22, respectively, with zero as mean and SD of 1.19 (Table 3). The maximum and minimum RP ability values were 4.27 and -1.58, respectively, with zero as mean and SD of 1.32. The mean of RP scores by sociodemographic variable and level is shown in Figure 2. The practices of reading the product labels as well as keeping the products out of the reach of children were related to a higher RP (Table 3). The practices of mixing some cleaning products as well as using a greater amount of them were related to a lower RP (Table 3).

**Table 3:** Rasch Model Fitness diagnoses for the Risk Perception Scale (RPS).

ITEM*	Difficulty (logits)	Standard error	Outfit	
			MNSQ	Z
Waste of cleaning products helps marine fauna.	-0.29	0.12	0.74	-2.2
Waste of cleaning products can contaminate groundwater.	-0.11	0.12	0.80	-1.8
Cleaning products are harmless.	0.11	0.11	0.88	-1.2
Waste of detergents helps to clean groundwater.	0.84	0.10	0.90	-1.6
It is possible to get clean surfaces using less detergent.	0.47	0.10	0.95	-0.6
It is necessary to use more detergent to get cleaner surfaces.	1.33	0.09	0.96	-0.8
It is important to read the cleaning products’ labels.	-3.22	0.39	0.99	0.1
Mixing different cleaning products is good to clean better.	1.35	0.09	1.01	0.2
Cleaning products can affect people’s health.	-0.07	0.12	1.07	0.7
Mixing cleaning products can be dangerous.	0.13	0.11	1.08	0.9
It is useless to read the cleaning product directions for use.	0.9	0.10	1.10	1.6
Waste of cleaning products reaches the ocean and affects marine fauna.	0.93	0.10	1.14	2.2
Cleaning products must be kept out of the reach of children.	-1.69	0.20	1.23	0.9
Children can ingest cleaning products by accident and get poisoned.	-0.66	0.14	1.39	2.3
Antibacterial detergents are better because they prevent diseases.	REMOVED			
Antibacterial detergents help some bacteria to get stronger.	REMOVED			

^*^ Items are listed in order of MNSQ. Last two items were removed due to their MNSQ values were higher than 1.5.

The OLS regression analysis for EA as dependent variable had an R^2^ of 0.0849 (F_5,678_ = 14.38, *p* < 0.001). Age and education had a positive impact on the EA score (Table 4). The OLS regression analysis for RP as dependent variable had an R^2^ of 0.2125 (Wald = 204.65, *p* < 0.01). Higher EA and education, as well as outside employment had positive impacts on RP score (Table 5). There was a significant difference between middle and high education odds ratio (Wald= 4.32, *p* < 0.05). Women who attended high school and college are twice as likely to have a higher RP as women without formal education or with only intermediate education (Table 5). A greater difference was found between participants with low education and those who attended college (odds ratio = 2.59) (Table 5). The RP scores by sociodemographic variables and levels are shown in the Figure 2.

**Table 4:** Results of OLS regression analysis for Environmental Awareness (EA) Scores as dependent variable (n = 678; R^2^ = 0.0849).

Environmental Awareness^*^	Coefficient^**^	t	*p*	Confidence interval (95%)^***^
Age (30–44)^a^	1.54	3.54	< 0.001	1.21–1.95
Age (≥45)^a^	1.93	3.33	0.001	1.31–2.86
Middle Education^b^	1.21	1.36	0.173	0.91–1.61
High Education^b^	1.66	3.74	< 0.001	1.27–2.16
Outside employee^c^	1.12	0.99	0.321	0.89–1.40
Constant	-0.79	-2.31	0.021	0.64–0.96

^*^ Environmental Awareness was measured in logits or the log-odds transformation of the probability of a response.

^**^ Regression coefficients are presented as exp(logits).

^***^ Confidence intervals were calculated using heteroscedasticity–correct standard errors.

^a^ Comparison group: < 29 years.

^b^ Comparison group: Low Education

^c^ Comparison group: Housewife.

**Table 5:** Results of OLS regression analysis for Risk Perception (RP) Scores as dependent variable (n = 678; R^2^ = 0.2125).

Risk Perception^*^	Coefficient^**^	z	*p*	Confidence interval (95%)^***^
Environmental Awareness Score	1.24	4.40	0.000	1.13–1.36
Age (30–44)^a^	1.23	1.68	0.092	0.97–1.57
Age (≥45)^a^	1.35	0.15	0.881	0.69–1.53
Middle Education^b^	2.00	5.48	0.000	1.56–2.58
High Education^b^	2.59	7.46	0.000	2.02–3.33
Employee	1.24	2.07	0.038	1.01–1.53
Constant	3.63	12.33	0.000	2.96–4.46

^*^ Risk Perception was measured in logits or the log-odds transformation of the probability of a response.

^**^ Regression coefficients are presented as exp(logits).

^***^ Confidence intervals were calculated using heteroscedasticity–correct standard errors.

^a^ Comparison group: < 29 years.

^b^ Comparison group: Low Education.

^c^ Comparison group: Housewife.

Discussion

Unlike previous studies on EA and RP that used Principal Component Analysis [74,75] and Factor Analysis [76–78], this study used Rasch Analysis [79]. Rasch Analysis allows for transforming ordinal data into continuous data, hence performing more robust statistical processes like regression analysis. However, our experience in this study confirmed what has been pointed out by previous researches [80] about the need to test Rasch Analysis during the survey design phase in order to guarantee its fitness to the model prior to application.

The EAS dealt with three issues: the sources of tap water, sewage management and vulnerability of groundwater to contamination. Only 51% of the participants knew that the actual source of tap water is groundwater (Table 2). What accounts for the remaining 49%? There are two possible answers. From a methodological perspective, it could be that half of the participants did not understand the concept of groundwater, which implies a bad item design. From a functional perspective, it is possible that the conceptual link between turning on the tap and extracting water from a well is hard to establish. It would mean that the groundwater concept is easier linked to a well than to a tap.

Regarding sewage management, even when women have a general knowledge about septic tanks, they mostly ignore the process that occurs there. This result could be related to low frequency of septic tank cleaning –even when water authorities suggest an annual cleaning [81] –or with a lack of information provided by the cleaning companies. Only 18% of participants thought that treatment systems turn sewage water into drinkable water, and 10% thought that sludge is used to produce fertilizers (Table 2). These low percentages are encouraging since they indicate that most of women are aware of the substandard sewage management in place, it also indicates a need for clear and true information about it.

The high percentage of women knowing that the aquifer is vulnerable to contamination could be explained by the increasing public concern about the environment [82] rather than from knowledge of the hydro-geo-ecological features of a karst system. This concern was expressed globally in the Brundtland Report of 1987 and with the United Nations initiative Decade of Education for Sustainable Development, 2005-2014. Around the time the data were collected, there were initiatives that included aquifer contamination as an issue of public concern [83,84]. The scope of this research does not analyze the impact of these local programs.

Almost two decades ago, Ronald Inglehart pointed out that the increasing concern for environmental issues was related to a change in the priorities of the societies, moving from a materialistic point of view to a post materialistic perspective, where meeting basic needs implies acquiring new concerns [85]. Detractors of Inglehart’s ideas maintain that higher environmental awareness is not related to higher wealth but to higher vulnerability to environmental hazards [86,87]. In this study, women with higher education obtained higher EA and RP scores (Table 4). Regardless of Inglehart’s assumption, it is likely that our results are related to better access to information and more diverse sources of social contact than with a different matrix of values. This situation could also explain the higher EA and RP scores in outside employees and women older than 29. It is important to take into account that more years of education mean more opportunities to develop the skills useful in responding surveys such as the one analyzed here.

Even when the global results of EAS show the overall effect of older age and more education, a closer examination of the results shows a lack of information about specific issues i.e. the disposal of sludge and gray water. This situation should be considered by environmental authorities and encourage the design of environmental education activities, which need to take into account the cultural diversity. The city could take School Education Boards to spread information to the public via the Schools.

Regarding the RPS, the diagnoses of its fitness to the Rasch Model forced us to remove two items, both related to increases in bacterial resistance associated with antibacterial cleaning products (Table 3), due to their high difficulty values. From the methodological perspective, it is necessary to rephrase those items to be useful in future studies. Notwithstanding, it is important to consider the impact of advertising that presents antibacterial products as a means of disease prevention and well-being [88]. Once those two items were removed, the items related to mixing cleaning products and the need to use more of each product to get better results, had the highest difficulty values (Table 3). Results show that the mixing of cleaning products, e.g. chlorine and hydrochloric acid is not perceived as a risk [89], and as our results show not related to a high RP score. By contrast, reading product labels and keeping the products out of the reach of children are practices related to a higher RP. Nonetheless, the fact of having a higher RP does not ensure that people actually perform these practices [90]. Studies have shown that most consumers do not read the cleaning product labels [91] or they think that those directions are exaggerated taking into account the product quality [92]. In our particular case, most of the cleaning products are bottled or bagged in small containers without labels; this is a common practice in small retailer stores. Store owners buy in bulk and retail small quantities to maximize gain [93]. Whether the labels are incomplete, useless, ignored or entirely missing, the environmental and health outcomes are the same: an increase of cleaning products in the sewage system, a considerable number of women affected by chlorine fumes and many children exposed to dangerous products.

Some studies about environmental risk perception or pro-environmental behavior supports the idea that young people with higher education and better health have a higher pro-environmental attitude [94], more perception about contamination, and are more willing to participate in pro-environmental activities [95]. Other studies point out that the related factor of lower earning opportunity does not necessarily mean a lower environmental RP [96] because the vulnerability of poor communities to environmental risks makes them more aware of this context [97]. Regarding health risks, higher education does not equate to a higher RP [98]. Despite the opposite conclusions of the cited studies, it is a fact that sociodemographic variables have an impact on RP [99], even when the results vary depending on the research purposes and techniques [100].

Our results show that more education and a formal employment have a positive impact on RP (Table 5). This result is consistent with previous studies [101]. However, as we said regarding the EAS results, it is necessary to consider the relationship between years of education and improved skills to answer questionnaires.

The last analysis we performed was to examine the link between EA and RP. According to our results, knowledge about water sources and sewage management in the city has a positive relationship with the cleaning product risk perception. This means that enhancing EA might improve RP and thus modify use patterns involving cleaning products. This would imply that less cleaning agents would pass to the aquifer and women and children’s exposure would be diminished. While the results indicate that a key to modifying behavior could be the implementation of educational strategies that increase knowledge about water sources and sewage treatment systems in the city of Mérida, a risk we propose three main ideas as communication strategy: a) the dangers of mixing cleaning products; b) the benefits of using fewer types of cleaning products; c) the benefits of using less product and achieve the same results. These ideas address the health and environmental risks derived from exaggerated cleaning products use. Even when the messages are simple, the principal obstacle to such a campaign would be that it opposes commercial interests [102] and deeply rooted practices linked to the use of products women are very familiar with [103]. Taking these factors into account, the effectiveness of risk communication activities could depend on the participation of all the stakeholders in the community [104] as well as on the testimonies of affected people [105].

Our results show the necessity to implement environmental education and risk communication. Hydro-geo-ecological conditions in Mérida will not change and septic tanks will continue to be used. This is also true for millions of households found in karst areas in the United States [106], France [107], Australia [108] and many other regions around the world [109]. The risk of contamination to groundwater by cleaning products will not decrease unless people change daily practices. Even though acquiring new information does not immediately lead to new behaviors, it is important to begin educating the community about water resources and sewage management. Increasing community awareness about the vulnerability of their water resources as well as the health risks associated with cleaning products is the first step to develop healthier and environmentally friendly practices and spaces.
